# Characterizing Smart Environments as Interactive and Collective Platforms: A Review of the Key Behaviors of Responsive Architecture

**DOI:** 10.3390/s21103417

**Published:** 2021-05-14

**Authors:** Ju Hyun Lee, Michael J. Ostwald, Mi Jeong Kim

**Affiliations:** 1 UNSW Built Environment, Faculty of Arts, Design and Architecture, The University of New South Wales, Sydney, NSW 2052, Australia; juhyun.lee@unsw.edu.au (J.H.L.); m.ostwald@unsw.edu.au (M.J.O.); 2 School of Architecture, Hanyang University, Seoul 04763, Korea

**Keywords:** responsive architecture, adaptive architecture, intelligent building, kinetic architecture, smart environment, sensing space

## Abstract

Since architect Nicholas Negroponte first proposed a vision of responsive architecture smart environments have been widely investigated, especially in the fields of computer science and engineering. Despite growing interest in the topic, a comprehensive review of research about smart environments from the architectural perspective is largely missing. In order to provide a formal understanding of smart environments in architecture, this paper conducts a systematic literature review of scholarly sources over the last decade, focusing on four related subjects: (1) responsive architecture, (2) kinetic architecture, (3) adaptive architecture and (4) intelligent buildings. Through this review, the paper identifies and examines *interactive* and *collective* behaviors in smart environments, thereby contributing to defining the properties of creative, smart spaces in the contemporary digital ecosystem. In addition, this research offers a means of systematically characterizing and constructing smart environments as *interactive* and *collective* platforms, enabling occupants to sense, experience and understand smart spaces.

## 1. Introduction

The rapid evolution of information and communication technology (ICT) has been a catalyst for transforming human-made environments into “smart” environments, which engage with users through sensors and digital devices. Architecture is no longer just defined by the “*hard*” walls of buildings, but by the responsive, adaptive, intelligent and kinetic [1,2,3] interaction between buildings and occupants [4,5,6], which in turn leverages the collected data by way of smart services [7]. New developments in electronics and materials have driven significant research into sensor networks for smart environments [8,9]. Furthermore, recent studies have addressed advanced smart environment applications using big data and the Internet of Things (IoT) [10,11,12]. Such research has, however, been conducted largely in computer science, electrical and mechanical engineering [7,8,10,12,13,14,15,16,17,18]. Consequently, a systematic understanding of smart environments from the perspective of architectural disciplines is largely unexploited. This is a critical knowledge gap, because the creation of physical smart spaces, from smart homes or offices to smart cities [4,7,10,19,20], requires a consideration of buildings. Thus, the underlying research question which this paper addresses is: “how has architectural research characterized and developed smart environments?”

To answer this question, this paper re-examines the behaviors and characteristics of *responsive architecture*, because many early ideas developed in this field are still valid for smart environments today. *Responsive architecture* is defined as a type of architecture that has the capacity to change its form in response to changing conditions [21]. It is, therefore, an artificial entity that reacts to data and information collected by a variety of types of sensors, and sometimes many hundreds of sensors [5]. The nature of the responsive architectural *behavior* may include physical actions (changes or movements) and adaptations in environmental services, such as lighting, heating and ventilation. For example, in Nicholas Negroponte’s “soft architecture machine” [22], *responsive architecture* is a physical environment exhibiting reflexive and simulated behaviors, and which is also a result of computation. Accordingly, the term *responsive* can refer to either adaptive or reactive activities, as well as *intelligent* ones, because the smart environment infers and presents diverse degrees of behaviors responding to different needs or circumstances [6]. In this way, recent architectural responsiveness uses advanced computing technologies integrating Artificial Intelligence (AI), robotics and “machine intelligence” [23].

Traditionally, architecture has been regarded as a socially-framed environment, but it has increasingly become an operable one through home automation [24]. Furthermore, since Negroponte’s characterization of responsive architecture, computing power and sensor networks have become faster, cheaper and smaller. The last of these, the reduction in size, has led to a situation where computing has effectively become invisible, ubiquitous and pervasive in physical environments [5,25]. Furthermore, the interconnectedness of spaces via global networks is integral to the concept of the smart city, where “everything” interacts in the IoT [6]. This highly flexible urban environment draws on, or mirrors, the 1960s vision of responsive architecture. From a micro perspective, viewing architecture as a skin—a continuation of our biological and sensorial system [26]—was once a core concept, but today we wear computers on our bodies and in our clothing, which extends their functionality to architecture and augmented environments. For example, Yoon’s “defensible dress” [27] treats space as an intimate extension of the environment of the body. Considering this range of *smart spaces*, this paper conducts a critical literature review on *responsive architecture* as a step towards characterizing smart environments.

As a second way of approaching the research question posed earlier, this paper expands the notions of *responsive architecture* and smart environments to consider their place in the wider “digital ecology” of *interactive* and *collective* platforms. In essence, an *interactive* and *collective* platform is the digital environment or system (hardware and software) where diverse computing or operating systems are executed, leading to architectural *behaviors* such as changing forms or services. The digital ecology is made up of the larger set of digital processes and products, which are facilitated by advanced ICT, including mobile and cloud computing, big data and IoT [28]. A digital ecosystem is therefore a large-scale ubiquitous system where digital actors—such as customers, partners and providers—and their interactive activities sustainably evolve [29]. In this model, smart environments can be regarded as a platform in a digital ecosystem that is full of digital services. In other words, smart environments are a dynamic platform which interacts with constantly changing data collected by ubiquitous sensor networks.

Consequently, this paper conducts a systematic literature review of smart environment research from an architectural perspective and presents its findings in terms of the larger digital ecosystem model of *interactive* behaviors (IBs) and *collective* behaviors (CBs).

- IBs are architectural behaviors involving transformation of physical forms or modification of environmental services that are visually apparent or perceptible in the environment. Rather than simply considering one-way service provisions from a building to a user, it encompasses two-way, continuously evolving, *interactive* responsiveness.
- CBs are sensing, thinking and controlling behaviors that *collectively* occur in an electronic (or digital) environment. Since most IBs are suggested and executed by CBs, IBs can be understood as a “product”, while CB is a “process”. In addition, sensing information is regarded as a trigger event resulting in architectural changes and/or context-aware services [5]. That is, it initiates CBs that develop IBs exhibited in the smart environment.

By considering both IBs and CBs, this systematic literature review can be used to examine the way that intelligent sensors and sensing information are designed to build smart environments.

Following this introduction, this paper defines the concept of responsive architecture to provide a foundation for the remainder of the research. After the methodology section, this paper presents the results of a literature review on (i) responsive architecture as well as three closely related topics, (ii) kinetic architecture, (iii) adaptive architecture and (iv) intelligent buildings. Across these themes, the literature review draws out a unique reading of the IBs and CBs of smart environments in the digital ecosystem. The paper concludes with a discussion about its contributions, limitations and future work.

## 2. The Concepts and Behaviors of Responsive Architecture

Negroponte, an architect and pioneer in the field of computer-aided design (CAD), described *responsive architecture* in his theory of “architecture machines” [3]. In this theory, advances in AI and the miniaturization of components collectively enable buildings to intelligently recognize inhabitants’ activities as well as to respond to their needs. As a result of this development, architecture can change its internal and external environments [21]. This concept is also found in Brodey’s “intelligent environments” [2] and Negroponte’s “soft architecture machine” [22]. Thus, *responsive architecture* can be defined as an environment which has embedded computationally-mediated responsiveness [1]. In the half-century since *responsive architecture* was first proposed, the ICT revolution, following Moore’s law, has enabled faster and cheaper machines than ever before. Consequently, architecture has already become adjustable to the changing needs of its inhabitants. Furthermore, it exists in the informative and interactive surroundings, or so-called “thick air”, which is presumed to envelop a building in an invisible sensor cloud, involving kinetic, sensing and environment-responsive systems [30].

Taylor and Lee [31] define *responsive architecture* as an inhabitable and operable environment as well as a collection of stimulus-response systems that create “real-time architecture”. Meagher’s [32] definition highlights the synchronous, changeable aspects of the environment and physical responses in buildings. Computers in the home already exhibit Negroponte’s operational and informational responses [22], which now allow complex architectural gestures and transformations. Interestingly, this smart architecture is not only realizing complex adaptive and real-time responsiveness, but it is also computationally networked as predicted [1]. Because it is co-evolving with its inhabitants, *responsive architecture* has been conceptualized as a living creature in a digital, connected ecosystem. From this point of view, responsive envelopes allow for co-evolutionary interaction between the inhabitant and environments [30]. Moreover, the physical and psychological boundary of space is blurred and mixed with the space of digital, virtual environments. This evolution of space is obviously of enormous architectural and behavioral consequence. Indeed, in traditional theory and praxis, a common question asks if architecture is “*more about particularization than generalization*” [33]. This can be addressed through reframing Negroponte’s concept of responsiveness, which evolves in line with advances in *computation* as well as in *materialization*. The first of these—computing technologies for individual responses—are widely studied in computer science [34]. In contrast the second—materialization—requires complex interaction via sensors in physical structures, which are more frequently addressed in architectural research.

Materialization, or the physical realization of a responsive environment, is a core issue in architecture. Although current advanced materials enable more flexible environments, they are still not free from the view that architecture is “hard”. Early pioneers of *responsive architecture* typically reacted to this situation in literal ways, creating “soft” surfaces, such as Eventstructure Research Group’s (ERG) inflatable plastic artworks in the 1960s. The exploration of such fragile or impermanent architectural materials has been a common thread in architectural design since that time, although most of this “soft” architecture is simply the reverse of “hard” architecture and is not a response to the deeper intention of the idea. Negroponte’s and Brodey’s soft architecture called for miniaturization of building components and their kinetics, which is arguably closer to the more recent practices of people such as Menges and Reichert [35] who developed a biomimetic responsive material system, using the material’s hygroscopic behavior and anisotropic characteristics, which constantly provides feedback and interaction with its surrounding environment. Such a material operates without any energy, mechanical or electronic control. It is part of a research field that is increasingly concerned with “intelligent skins” [36], “smart materials” [37], “shape memory alloy (SMA)” [38], “thermobimetal” [39] and “nanomaterial” [40].

Like human behaviors, smart environments have specific ways of behaving in response to particular conditions. Considering artificial responses in interactive artwork for example, Lee et al. [6] identified two important reflexive behaviors (tangible interaction and embodied response) and two simulated behaviors (ambient simulation and mixed reality). The first—reflexive behavior—involves self-organizing controllers, recognizing mood and the enhancement of mutual involvement [22], which Negroponte historically acknowledged were difficult to visualize. Emerging architectural technologies not only enable complex, personal non-linear interactions [41], but also information dense, real-time interactive and constructive responses [27]. To develop this responsive artificiality, architecture incorporates sensory data into a central inference system to interpret human needs and/or environmental contexts, by way of a sensor-based context-aware system [5]. The intelligent system then suggests appropriate architectural responses that are distributed into transformable building components [42] or smart materials [37]. These reflexive behaviors transform the built environment from a collection of *static* objects into a “smart” system of dynamic and interactive built forms [41]. The resulting transformable architecture could be called a “machine” [43], but it is not just mechanistic. Reflexive behaviors can include the mechanical and biological properties of building components and materials, along with the “robotic” ones [44].

While reflexive behaviors have been difficult to implement, simulated behaviors are more common and easy to create, as suggested in the concept of the “*simulatorium*” [22]. Since first being proposed, this simulation has used various visual devices, from Sutherland’s head-mounted display (HMD) [45] for virtual reality (VR) or augmented reality (AR), to eyewear such as the Google glass [46] and screen projection-based displays such as the CAVE Automatic Virtual Environment (CAVE) [47] and iDome [48]. Simulated responses are also integrated into mobile computing, developing mobile augmented reality (MAR) [49]. The CAVE, for example, supported a graphical VR system using an off-axis perspective projection [47]. The simulated behavior in CAVE is calculated corresponding to the viewer’s position with respect to the locations of the walls. As another example, iDome is a panoramic visualization system within a 180-degree fiberglass dome surface, that uses a high-definition projector and a spherical mirror [48]. Using a track ball, the user rotates the projection, and the multi-channel sound is adjusted according to the user’s viewing position. The CAVE highlights responsive visualization, while iDome demonstrates ambient sound responses in interactive VR. The fully immersive sensory experience, including both the visual and the auditory, is essential to creating ambient simulations. The analytic stage of the literature review in the following sections identifies further key architectural behaviors including tangible and intangible actions or services.

## 3. Research Method

Adopting a systematic literature review approach [7,10,50], this paper examines scholarly works, focusing on refereed journal papers that have been published in the last decade (2011–2020). This review is targeted to a defined theme—smart environment research in the architectural disciplines—and four keyword combinations: “responsive architecture”, “kinetic architecture”, “adaptive architecture” and “intelligent building”. These four can all be traced to some of the earliest research in smart architecture [1]. Whilst a keyword search using “smart environment” results in many hundreds of articles in computer sciences and engineering, the four domain-specific keyword combinations narrow the scope of the search to the research question framed at the start of this paper.

Three academic databases were used for the systematic literature review: *ScienceDirect* (https://www.sciencedirect.com/, accessed on 16 February 2021), *SAGE journals* (https://journals.sagepub.com/, accessed on 16 February 2021) and *Taylor & Francis Online* (https://www.tandfonline.com/, accessed on 16 February 2021). These three encapsulate the majority of indexed journals in architecture domains. Using the abstract, title and keywords fields, the four keyword combinations were used to identify relevant research. After the initial search, a review of content was undertaken to manually exclude irrelevant or duplicate references. At the conclusion, a final set of 226 research papers was collected from the three online databases. This was subdivided into four datasets corresponding to the keyword combinations.

Table 1 summarizes the general characteristics of the four datasets. The last set, “intelligent building”, is the dominant one, accounting for 173 articles. In contrast, the keywords “kinetic architecture” and “adaptive architecture” identified only 14 articles each. The top three journals in each dataset are also listed in Table 1. Both *Architectural Science Review* and *International Journal of Architectural Computing* published the most articles on “responsive architecture” (four articles each). *International Journal of Architectural Computing* also published the most articles on “kinetic architecture”. In contrast, *Energy and Buildings* published the most articles on “intelligent building” (30 articles) and *Intelligent Buildings International* was the second in this dataset (23 articles). In the “intelligent building” category, multiple journals are concerned with energy and environment, for example, *Energy and Buildings*, *Building and Environment*, *Renewable and Sustainable Energy Review*, *Applied Energy* and *Energy*. Whilst the last dataset includes many energy- or computing technology-focused studies, *Intelligent Buildings International* deals with a variety of topics including AI, biomimetics and biophilia [51,52].

Within the complete set of 226 articles, a search was then undertaken to identify *interactive* (IBs) and *collective* (CBs) architectural behaviors. Within this review it was apparent that the IBs and CBs are only rarely divided, with most being integrated into the architectural system where they work together. In the literature, however, IBs tended to refer to architectural responsiveness (actions or services), which is often visible or perceptible, while CBs were related to sensor networks (sensing), intelligence (thinking) and control systems (controlling), which are usually hidden. Of course, a user’s direct control would be an IB, but it is rarely addressed in the literature. In this way, the following section reviews the research papers of the last decade with a focus on IBs and CBs in architecture.

## 4. Findings

### 4.1. Responsive Architecture

The dataset for “responsive architecture” contains 25 research articles (Table 1). There is, not surprisingly, some overlap between this first theme and several others, with some articles in this set also referring to adaptive building systems [53,54,55,56,57] and kinetic systems [55,58,59,60]. A few studies also address aspects of embodied intelligence and performance [57,61,62], which are related to “intelligent buildings”. Therefore, in this section the 25 works are examined regardless of overlaps, whereas in the next sections, only those papers relevant to the other keyword combinations are considered.

Importantly, three articles identified in this set offer detailed literature reviews on related topics. For example, Megahed [61] categorized the hardware and software components of responsive architecture into four systems: material, informational, processing and behavioral. Meyboom et al. [63] examined literature on mechatronic interactive systems that respond both to occupation and environment, addressing “*architectronics*”, the combination of architecture and mechatronics. Bitterman and Shach-Pinsly [64] reviewed the technologies, objectives, problems and obstacles of the smart home, and discussed their future implications. The smart home, as a dynamic smart environment, can respond to changeable and personalized human and social needs. As a result, it enables the formation of a smart community in the digital ecosystem. Through their review Bitterman and Shach-Pinsly highlighted several factors such as improving security and saving energy.

Multiple studies on responsive building skins have also been produced in the fields of architecture and construction. Loonen et al. [57], for instance, described climate adaptive building shells (CABS) that improve environmental, societal and economical performance. The responsive behaviors of CABS are adjusted extrinsically using three elements (sensors, processors and actuators), recording four properties of CABS (thermal, optical, airflow and electrical). In contrast, solar shading devices provide a unique response using smart materials including colour-changing, photovoltaic (PV) and shape-memory materials (SMMs) [65]. Barozzi et al. [59] conducted an assessment of multiple adaptable envelopes and façade shading systems that reduce energy consumption. Al-Masrani et al. [66] also reviewed dynamic shading systems, highlighting design elements and platforms as well as evaluation strategies. The geometric-based analysis of dynamic shading systems addresses model design intricacy (geometric strategy, kinetic complexity, motion types and typologies), while the performance-based analysis considers the impact of operational functionality on the built environment (design criteria, control strategies and energy situation).

#### 4.1.1. IBs of Responsive Architecture

Meyboom et al. [63] maintain that Le Corbusier’s design for the 1958 Philips Pavilion, (“*Poème Electronique*”), which integrated music and visual display with the building, was an early example of an IB in architecture. Michael Webb’s 1960 *Magic Carpet* presented the IBs of a dynamic fluid and air jet environment supporting a body in space. Jean Nouvel’s responsive screens in the 1988 *Institut du Monde Arabe* in Paris were also intended to use light-sensitive diaphragm devices. IBs and responsive facades of these types remain a topic of study in recent years [63]. For example, interactive kinetic media facades now use Delta robot kinematics (e.g., piston motion, radial motion) and LED effects [60]. Adaptive solar facades also use soft pneumatic actuators that bend under applied pressure [67]. These *mechatronic behaviors* are responses to the changing needs of the inhabitants as well as changes in the environment [32,63]. Responsively, the behavioral systems transform architectural form, shape, colour or character through actuators [61].

Climatically responsive IBs are a common category in the research. They have been widely used in shading systems in contemporary architecture [53,55,56,57,58,59,62,63,65,66,68]. In façade design, high quality luminous environments using this sort of IB not only satisfy human visual needs, but also develop a sustainable and climatically responsive architecture [62]. There are three broad types of solar shading devices mentioned in this research: membranes (awnings, curtains), shutters (folding/sliding panels) and kinetic devices (louvres and other kinetic façades) [65]. In addition, *climate-responsive behavior* supports improved thermal performance and comfort in a building [62]. A customized dynamic architectural façade or CABS [53,57] can respond to thermal, optical, airflow and electrical behaviors. Micro adaptation of CABS is typically limited to changes in thermophysical, opaque optical properties and the exchange of energy, while its macro adaptation can involve subsystems of the building shell itself as well as movement of the entire façade or the building as a whole [57].

In recent years adaptive origami-based modular structures [55,56,58] have been proposed as a means of enabling architectural responsiveness to light, thermal and acoustic conditions. The *origami-based behavior* is typically a sensing and actuation system much like the other climate-responsive building skins [56]. In contrast, a kinetic solar skin using a thermo-mechanical SMA actuator can exhibit similar *self-organizing behavior* to origami folding patterns [55,58]. In this case, the responsiveness can be understood as a *material-based behavior* that enables a lightweight, motorless and silently operable building system [58]. Such climate-responsive IBs of adaptive façades have implications for architectural customization, sustainability and aesthetics [56] as well as building performance [66]. Many of these interactive building skins rely on *mechatronic behaviors* based on kinematics, while some solar shading devices use smart materials instead (e.g., property-changing smart materials, energy-exchanging smart materials) [65].

Research in the dataset for responsive architecture also confirms that the optimization of materials not only impacts on architectural robustness and performance, but also determines its responsive behaviors. Thus, many articles describe the adaptation or development of appropriate materials [54,56,58,59,65,69,70,71,72,73]. Smart materials have been developed for material interaction integrating sensing and actuation into the fabric of architecture [65,71]. In contrast, *biomimetic behaviors* responding to environmental changes use, for example, hygromorphic materials [54,70,72] or wood-moisture relations arising from the shrinkage or swelling of wood [72]. This naturally responsive mechanism is not only predictable and reproducible but also reversible [70]. *Biomimetic behaviors* can be further developed using responsive programmable biofunctionality and microscale patterning using a fluidic system (glaze chemistry, texturing and geometry) and light-responsive ceramic bio-tiles (fluorescent or colour-changing) [73]. Such examples enable metabolically independent movement or materially-embedded responsiveness in the smart environment.

The IBs of responsive architecture can accommodate both spatial adjustments and qualities. Spatial adjustments involve linear displacement of a partition, increasing available surface area, while spatial qualities are related to geometry, colour and lighting, acoustics and ventilation [43]. Such IBs can arise from bio-inspired elastic kinetic shading systems, soft textile shadings (changes of bending curvature), changes in length of the membrane strip, a self-supporting shell structure or the intrinsic capacity of wood (wood cones) [59]. Unexpectedly, although natural interactions and *simulated behaviors* can be a key behavior in responsive architecture [6], they were rarely identified in the dataset for responsive architecture. Natural interactions, like embodied interactions, are generally developed using motion tracking, gesture recognition, emotional detection, facial expression identification and speech processing [64]. This embodied IB can support healthcare-related functions (e.g., early detection, diagnosis, monitoring and documentation, prevention, treatment, alleviation of disease, rehabilitation, wellness management and motivation) [64].

#### 4.1.2. CBs of Responsive Architecture

CBs of responsive architecture typically correspond with IBs in an architectural system because a certain behavior is triggered in response to a particular situation or stimulus. For example, to appropriately manage the *climate-responsive behaviors* of a building façade, preset programming is typically used to track the sun movement [63,67]. Furthermore, *sensing behaviors* capture temperature and humidity, sun tracking and daylight harvesting, light levels, movement and local environmental conditions [59,63]. *Origami-based behavior* is also based on these *environmental sensing behaviors* using a network of micro-sensors, detecting, for example, an a priori defined noise threshold (interior) and optimal light conditions (exterior) [56]. These sensor data and embedded computational elements then regulate the quality of light [32]. Temperatures and optimization criteria—including energy-related indicators and fluid-dynamic analysis—can also be used for this CB [53]. Energy-related indicators include glare probability, daylight and illuminance uniformity, and factors of external view. In addition to sensor-integrated automation, *climate-responsive behaviors* can be triggered by user interaction with an app-based remote control [58,67].

In general, there are two types of *automated control behaviors* in the research in this dataset: intrinsic and extrinsic controls. Extrinsic controls deal with distributed CBs via embedded computation in local processors, and/or centralized CBs triggered by a supervisory control unit to fulfil global target values [57]. In contrast, intrinsic controls tend to be self-adjusting, or automatically triggered by environmental stimuli (e.g., temperature, relative humidity, precipitation, wind speed and direction, solar radiation, cloud cover or CO_2_-level) [57]. In addition to environmental and infrastructure sensing, wearable sensors can be implemented for natural interactions based on real-time processing and data transmission via wireless body communication networks [64]. Park [60] furthermore identified various aspects of CBs, including user engagement (active and passive), number of users, input devices (e.g., sensors, Radio frequency identification (RFID)), local and global network, expressive, responsive (linear) and interactive (single loop) intelligence. A special case is “*sensponsiveness*”, where ambient intelligence imbues space with cognitive capacity [43].

The automation and control aspects of CBs (digital electronics) can have a significant impact on the performance of a building. They include control strategies and scenarios (e.g., open-loop or closed-loop systems, single- or multi-variable systems), controlling technologies (e.g., self-sensing and self-actuating technology) and controlling algorithms [66]. Control strategies can further be categorized into single variable-man, multivariable-man, multivariable automatic and multivariable heuristic controls [61]. *Intelligent control behaviors* address these control strategies and mechanisms with feedback. Control mechanisms and feedback simply consider both open-loop and closed-loop systems. An open-loop system responds to a signal in a predefined way, while a closed-loop system uses a feedback system [61]. In this way, advanced lighting controls can moderate levels of natural and artificial illumination, using digital dimming ballasts and programmed and user operable controls [62]. In summary, a control system or inference system translates sensor signals from user interaction or climate change into actuation commands. Actuators then produce reactions in the smart environment, converting energy into movement. In this process, the interactive or collective stimuli are captured by sensors and elaborated by the computation of the control system [59].

In contrast, as discussed in the previous section, smart materials have self-powering and self-actuating systems. For example, hydrogel biomaterials can innately respond to pH, glucose, oxidants, antigens, enzymes, ligands, temperature, pressure and light [73]. “*Hygroexpansion*” is also based on moisture-induced opening and closing [54]. Thus, CBs are rarely exhibited in the sort of smart environments enabled through the use of smart materials. Smart materials can also be pre-programed [54] or use shape-changing rules integrating material transformation into shape computation [72]. Thus, this *self-actuating behavior* is like an SMA’s *self-organizing behavior* [55,58], which is a research topic that has not yet been explored in the literature.

### 4.2. Kinetic Architecture

#### 4.2.1. IBs of Kinetic Architecture

The phrase “kinetic architecture” typically refers to a mechanical and movable structure or organism, as architecture is often described metaphorically in biological terms [31]. Developments in robotic technologies as well as digital design processes have allowed architecture to become more flexible and adaptable in response to changing needs [74]. Ramzy and Fayed [44] provides a historical, evolutionary overview of kinetic architecture, from early primitive kinetic systems (e.g., pivoting, sliding openings) to advanced kinetic systems using AI. Twentieth-century kinetic systems already adopted a variety of mechanical, lightweight and flexible structural systems to create movable designs, while recent kinetic systems developed have employed computerized systems to create intelligent architecture [44]. Holden [75] provides a unique way of looking at this dataset, through an analysis of Jean Tinguely’s kinetic movements. Tinguely’s interactive constructions and artworks in the 1960s provided a different model of the relationship between art and architecture. Tinguely’s kinetic structures, or “meta-mechanicals”, can be regarded as participatory art, presenting an operative concept in both art and architecture. His large-scale kinetic (also interactive and collaborative) projects provided new types of platforms for cultural activity in the urban environment. For example, *Dylaby* using scaffold-like frames (1961) represented the unpredictable movement of the audience as well as the kinetic movement of architectural elements [75]. *Tiluzi* (1967) also exhibited the “regulated movement of the Ferris wheel” and the “erratic movement of a long circuitous slide or ramp” [75]. Drawing from this work, it is possible to identify kinetic systems as those which can automatically fold, slide and expand in both size and shape, exhibiting various IBs such as intelligent circulation (vertical and horizontal), environmental response (e.g., revolving buildings and responsive roofs) and flexibility for inner spaces (e.g., movable partitions) [44].

Despite such definitions drawn from art, kinetic architecture remains a contested concept in architecture, and there are multiple, sometimes conflicting terms and typologies in use [74]. One view is that kinetic systems embedding computational intelligence in architecture to enable it to be “adaptable, collapsible, deployable, enabling, evolutionary, flexible, intelligent, kinetic, mobile, performance-based, reconfigurable, responsive, revolving, smart, transformable, and transportable” [74] (p. 132). “Adaptable” structures (e.g., movable-wall systems) are easily altered or modified to meet different social functions, while “deployable” structures are capable of automatic configuration changes. “Intelligent” structures learn and respond to the information collected from the exterior or interior environments and “performance-based” structures use digital technologies to support the environment, users and society [74]. That is, each variation in terminology represents a specific IB of kinetic architecture.

The kinetic mechanisms identified in this literature also include two types: spatial-real movement and non-spatial-material deformation [74]. The former presents basic movement—folding, sliding, rolling, expanding and transforming—by changing the axis, strength and direction of kinetic elements. The latter uses smart materials driven by their molecules’ ability to change form, function or appearance [74]. In contrast, Ramzy and Fayed [44] argue that kinetic systems may be classified into four types: (i) skin-unit systems, (ii) retractable elements, (iii) revolving buildings and (iv) biomechanical systems. Skin-unit systems include responsive and interactive facades, flare skin and movable louvers. These systems typically support *climate-responsive behaviors*, although they can also respond to other information, such as pedestrian movement. The *mechatronic behaviors* using deployable kinetic structures are clearly seen in “retractable elements”, which fold or expand creating entire architectural element (roofs, walls, floors, etc.). Whilst “revolving buildings” respond to wind-power or solar energy, “biomechanical systems” using embedded or dynamic kinetic structures adjust themselves in respond to inner or outer forces [44]. The last category, *biomimetic behavior*, is mostly reliant on smart materials as described in the previous section.

The most dominant IB in the kinetic architecture literature is a *structural adaptive behavior* using folding mechanisms [76,77,78,79,80,81,82]. For example, linkage mechanisms for kinetic architecture include Watt-I linkage as a one degree-of-freedom (1-DOF) mechanism [76], spherical linkages [78,82] and a two degree-of-freedom (2-DOF) 8R (revolute joint) linkage [79]. The Watt-I “finger linkage” used in robotic, anthropomorphic fingers has been adopted for a convertible stadium roof structure, providing a wide range of structure flexibility and shading options [76]. Spherical linkages with Miura-ori folding mechanisms have been used to create deployable surfaces [78,82]. Their *translational motion behavior* uses scissor-like structures that develop the target curvature by changing the length of the bar and creating foldable assemblies [78]. Lastly, from the Bennett linkage using a 4R spatial linkage, Korkmaz et al. [79] suggest a 2-DOF 8R linkage for transformable hyperbolic paraboloid (“hypar”) structures. The 2-DOF system allows various configurational structures and wider form flexibility, responding to dynamic and constantly changing activities. This kinetic, *structural adaptive behavior* is most often applied to roof structures [76,77,79,80].

Research has also proposed the development of lightweight roof oculus structures to integrate two cooling strategies for desert climates: evaporative cooling (day) and radiative cooling (night) [77]. The prototype for this roof oculus uses a slab as a thermal mass, storing coolness which influences geometric kinetics (constricting or releasing the opening). In contrast, another example proposes a pliable structure based on curved-line folding and *origami-based behavior* (the combination of folding and bending paper) [81]. The pliable structure can present origami-like *self-organizing behavior* [55,58], while curved-line folding creates a more complex 3D shape through a curved crease as well as an elegant folding motion. This example uses both *material-dependent behaviors* and Finite Element Analysis (FEA) to enable its kinetic and structural behavior [81]. Developable surfaces are also explored in the literature for their capacity to exhibit such behaviors [78,81,82].

The last IB of kinetic architecture develops ASF using a kinetic PV shading system [83]. This type of ASF is described in the “responsive architecture” dataset as *mechatronic behavior* [62,67]. In contrast, Jayathissa et al. [83] address performative design environments that enable a solar radiation model, PV electricity production, a building energy model, a daylighting model and their optimization. In summary, past research in kinetic architecture typically deals with various aspects of kinetic hardware and its *structural kinetic behaviors*.

#### 4.2.2. CBs of Kinetic Architecture

Kinetic systems in this dataset are typically associated with sensing and actuation systems that are largely reliant on *environmental sensing behaviors*. One example, a kinetic roof structure, uses “DHT-22 temperature and humidity” sensors and a cable-driven actuator network [77]. However, the second dataset barely describes any other CBs, and mostly just highlights the physical changes or movements of architectural components. One exception [84], like [66] in the previous dataset, describes the use of control scenarios. It argues that metamorphic architecture can be developed through scenario-based design, addressing problems, activity, information, interaction and usability scenarios [84]. “Activity”, which is the transformation of a current setting into a new configuration, and “interaction” scenarios are closely related to IBs, while “information” and “usability” scenarios support the development of effective CBs.

### 4.3. Adaptive Architecture

#### 4.3.1. IBs of Adaptive Architecture

Adaptive architecture “has the ability to alter its physical properties (form, shape, colour, texture, acoustic, porosity, etc.) in a predefined/programmed/designed way to adapt to changing external and internal environmental stimuli (temperature, relative humidity, precipitation, wind, sound, solar radiation, CO_2_-level, etc.), user activities and needs, and social contexts” [85] (p. 557). This definition emphasizes the synergies of overlaps with the broader concept of responsive architecture. The definition also stresses the importance of pre-programed elements which facilitate “adaptivity” or “adaptiveness” in architecture. Such elements often include smart materials, and it is not surprising that one of the papers in the third dataset, Abdullah and Al-Alwan [86], contains a review of past research into smart material systems that can create adaptive architecture. Their survey classifies smart materials into two types (property change and energy exchange) and smart material systems into three types (passive, active and hybrid). Since a smart material has multiple functions, including sensing changes that trigger actuation, its classification considers the way it responds to stimuli. Furthermore, they argue that combining different types of smart material systems can produce a higher level of adaptivity in architecture. Thus, *biomimetic behaviors* of smart materials play an important role in adaptive architecture, using smart materials’ hygroscopic behaviors [86,87,88] and even plants’ biological adaptation [89].

The *hygroscopic behavior* of wood, in response to changes of relative humidity (e.g., false ceiling opening), is one example of an adaptive hygrothermal comfort system [87]. This sort of IB, which was discussed in the previous section, has additional pertinent qualities when considered under the heading of adaptive architecture. Both structures of plywood and “unplywood” using active and passive layers are examined in the literature [87]. The bending reaction of wood bilayers is also developed in a multi-element wood-GFRP (glass fibre reinforced polymer) bilayer [88]. The wood and wood-hybrid bilayers can accommodate controlled and reversible shape changes in reaction to relative humidity. Furthermore, the *hygroscopic behavior*, the properties of curvature (e.g., specific sizes, shapes and aspect ratios), can be controlled or designed to achieve a particular outcome [88]. A different approach is to use organisms’ *survival*, *evolutionary* or *natural behavior* in a building façade system [89]. For example, plants in a façade can react to light, temperature or water changes and support building performance (energy saving) as well as occupants’ comfort levels at the macroscopic and microscopic scales. Like some other smart materials, there is no clear CB in the plant’s biological adaptation, because an organism’s shape, size, pattern or structure naturally depends on its surroundings.

As it was for kinetic architecture, the dominant IB of adaptive architecture is a *structural adaptive behavior* [90,91,92,93]. The *structural adaptive behavior* relies on a controllable dynamic system consisting of sensor-actuators, structural elements and skins. It then develops a transformation from a load-bearing behavior to a dynamic one [94]. These dynamic interactions between occupants and buildings enable, for example, the reduction of energy consumption and emission rates as well as occupants’ comfort [94]. As for the adaptive building skin, the load distribution of structural elements is regarded as an IB of intelligent machines [90], supporting real-time activation as well as *self-learning behaviors*. Building components identified in past research include façade elements, canopies and other structural features, where load distribution parameters and spatial parameters are used to adapt to unpredictable forces [90].

In addition, an interactive and optimized behavior can be applied to deployable building structures using tensegrity and scissor-like systems [90,91]. In such systems a lightweight linkage structure provides a generic 1-DOF, an effective crank-slider mechanism and a n-bar linkage with direct or cable-driven actuation [90,91,92]. Such a system is also flexible, expandable and controllable through modularity and actuation. In this way, structural elements become reconfigurable, and structures become self-erectable [90,91,92]. Structural reconfigurations involve morphological changes, manipulation and locomotion [92]. When considering all of these features, Phocas et al. [91] and Christoforou et al. [92] identified multiple IBs of *reconfigurable architecture*, such as optimizing the performance of a PV roof; optimizing distribution of structural and minimizing aerodynamic loads; optimizing occupants’ comfort by adjusting ventilation and lighting; improving space utilization; harvesting sun, wind and rain water; removing snow from roofs and producing unique aesthetic effects. Pruitt et al. [95] also describe historical ideas about the *comfort-ensuring behaviors* of adaptive architecture (e.g., climate-responsive façade design and mechanical ventilation systems).

#### 4.3.2. CBs of Adaptive Architecture

Intelligent CBs, such as self-learning algorithms or artificial neural networks (ANNs), created by a genetic algorithm develop various *structural adaptive behaviors*. Active control systems also use a database (knowledge) of pre-calculated equilibrium solutions. In this way, *structural adaptive behaviors* not only enable real-time measurement and optimization of the environment, but also improve their adaptation processes over time through *self-learning behaviors* [90]. In addition, an irregular self-bearing structural system, such as a tensegrity-membrane structure, can be used for wind-adaptive architecture, changing the aerodynamics of a building [93]. CFD simulations can be used to model its potential CB.

In another example, the biofeedback-driven system “*ExoBuilding*” provides the immersive effect of adaptive architecture (specific interactive effects), using recent technologies such as pervasive computing and a tent-like fabric structure [96]. The system presents biofeedback in an immersive, evolutionary fashion, in terms of *embodied behavior*. Thus, its CBs address the capturing of various types of personal data (e.g., location information, activity, social networking data, reactivity around one specific type of personal data and physiological data), controlling its central flexible spine via servomotors [96]. In addition, AI (e.g., ANN, multi-agent system (MAS) and EM algorithm) can be used for lighting control in smart cities [97]. Its goal is efficient energy management, detecting foot traffic patterns, managing ANN to predict consumption from light intensities and estimating energy consumption. Thus, the intelligent street lighting system collects information such as pedestrian and traffic flow and weather data [97]. Such methods are also suggested for climate-adapted architecture for energy saving [98], which is closely related to the theme in the following section.

### 4.4. Intelligent Building

The title “intelligent building” can refer to an “automated building”, a “smart building” or various types of “green building”, including energy-efficient and low-carbon buildings [99,100]. Whereas the previous datasets and themes in this chapter have had multiple potential applications, the intelligent building is most often linked to energy, sustainability and comfort [101]. A smart building operates in a way to minimize energy consumption through automation of operations as well as to ensure its occupants’ comfort (interactions between occupants and buildings) [99]. Mofidi and Akbari [99] identify six *intelligent behaviors* of the built environment: (i) indoor environment monitoring, (ii) communicating with occupants, (iii) energy-related decisions using energy management systems (EMS), (iv) energy-related actions using energy management and control systems (EMCS), (v) a learning capability and (vi) proper communication to the grid [99]. Dong et al. [102] reiterates several of these, echoing the importance of both energy saving and occupant comfort (e.g., thermal comfort, visual comfort and indoor air quality) in the smart building, although their systemic review is limited to sensing systems for indoor environmental control. Furthermore, Nguyen and Aiello [103] present building energy and comfort management (BECM) systems that satisfy the occupants’ comfort while reducing energy consumption. Collectively, most research about “intelligent buildings” is ultimately focused on energy or comfort.

#### 4.4.1. IBs of Intelligent Buildings

The most dominant IB found in the intelligent building literature is concerned with either “*energy efficiency behavior*” [104,105,106,107,108,109,110,111,112] or *“energy saving behavior*” [100,102,103,105,106,112,113,114,115,116,117,118,119,120]. Ding et al. [100], for example, identify research trends in *building energy saving* using a text mining methodology. In their survey, heating, ventilation and air-conditioning (HVAC) systems, energy technologies and lighting systems have been the major topic of 1600 articles on energy saving from 1973 to 2016. Thus, *HVAC and lighting behaviors* should be a fundamental IB of intelligent buildings. The most common topics of recent articles (2010–2016) are green building envelopes, building retrofitting, system operations and building information. These reflect recent academic interests in the integration of a solar energy system or a life-cycle management system using building information modelling (BIM) [100]. Nguyen and Aiello [103] offer an alternative definition, *energy intelligent buildings*, which refers to “buildings equipped with technology that allows monitoring of their occupants and/or facilities designed to automate and optimize control of appliances” (p. 247).

In contrast, there are two dominant *comfort-ensuring* IBs: “*thermal comfort behavior*” [102,104,107,108,117,118,119,121,122,123,124] and “*visual comfort behavior*” [102,106,117,123]. In comparison with many exterior-oriented behaviors identified in the previous sections of this paper, research about “intelligent building” often examines multiple indoor comfort-ensuring IBs and CBs including indoor daylight [104], environmental quality (IEQ) [105,125], thermal comfort [121,122] and air quality [117,126], navigation [127], positioning [128] and even indoor electrical IoT [128]. A comprehensive review by Mofidi and Akbari [99] of intelligent buildings categorizes six EMS topics: (i) occupant comfort conditions, (ii) occupant productivity, (iii) building control, (iv) computational optimization, (v) occupant behavior modelling and (vi) environmental monitoring and analysis [99]. As for the first two *comfort-ensuring IBs*, an intelligent EMS not only addresses thermal comfort, lighting and daylighting, visual comfort and indoor air quality (IAQ), but also supports the occupants’ productivity and well-being. The Leadership in Energy and Environmental Design (LEED) certification program, the WELL building standard and the Building Research Establishment Environmental Assessment Method (BREEAM) can be used for the productivity standards and guidelines [99]. Interestingly, whilst the second dataset, *kinetic architecture*, was largely focused on IBs, articles in the last dataset are more commonly concerned with CBs than IBs. This characteristic is to be expected, because the term, “kinetic” strongly indicates a physical movement in a product, whereas the term, “intelligent” is related to “thinking” as the process in the operations of smart environments.

#### 4.4.2. CBs of Intelligent Buildings

First of all, for an intelligent building to achieve *energy savings* it typically controls lighting, HVAC and “plug loads” (energy used by appliances), depending on occupant presence and behavior [103]. For example, Aftab et al. [116] present an occupancy-predictive HVAC control system using embedded system technologies (e.g., real-time occupancy recognition, dynamic analysis and prediction of occupancy patterns and a model of predictive control). The real-time occupancy recognition is achieved using video-processing and machine learning (ML) techniques, while the HVAC system is supported by a real-time building thermal response simulation using EnergyPlus [116]. A recent cloud-based system for energy information management also monitors, analyses and controls the energy use of a building. The cloud forecasting system uses a hybrid AI model—seasonal autoregressive integrated moving average (SARIMA) and metaheuristic firefly algorithm-based least squares support vector regression (MetaFA-LSSVR)—to characterize energy usage patterns, and to predict energy demand in real time [113]. To improve energy efficiency and thermal comfort, a model predictive control (MPC) design has adopted a tuning methodology that takes account of process disturbances, temporal parameters and weights on the objective function [119]. *Energy-optimizing CBs* are fundamentally involving such intelligent control systems that consist of numerous sensors and computational intelligence.

A BECM system evolves with *intelligent control CBs* based on AI [129]. Thus, multiple articles in this last dataset examine AI [113,121,129,130,131] and ML [106,113,129,132]. Panchalingam and Chan [129] conduct a literature review of research on AI technologies for smart buildings, focusing on nine topics: ML, natural language processing, deep learning, pattern recognition, machine vision, expert systems, ANN, fuzzy logic and genetic algorithms. Interestingly, *energy saving behaviors* in their survey largely adopt ML, supported by ANN, fuzzy logic and genetic algorithms. These computing systems also support *structural adaptive behaviors* (described in the previous section).

Multiple expert systems for reducing energy consumption have been developed at various scales. For example, an ANN control model for optimized distribution and heat trading effects can be used for responding to occupant characteristics, optimizing supply air condition and maximizing energy cost savings [122]. A two-layer ANN is also used for inferring occupancy counts from existing ICT system data [133]. Non-linear models based on fuzzy logic and ANN have been applied to predict electricity consumption and develop energy efficiency strategies [109]. As such, the intelligent building can be integrated using a micro-grid based on renewable energy resources (RERs) and energy cost coefficients (ECC) [134]. An energy-efficient outdoor lighting control system is also based on an expert system that uses knowledge-based rules for real-time control and monitoring function [130]. In this context, Aduda et al. [135] suggest the creation of an “*energy and comfort* active building” using a MAS, which interacts with electrical smart grids. Its EMS involves four levels of informational flows (communications): use level, building management level, agents/agent platform-utility grid and utility grid side communications [135]. A building EMS can also use power line communication [120]. Importantly, *energy-optimizing behaviors* are based on real-time occupancy information about preferences, patterns or use and activities [103]. Again, in order to recognize occupants’ activities, energy intelligent buildings adopt various technologies and approaches: logical inference from sensor data, ANN, fuzzy-logic-based incremental synchronous learning (ISL) systems, Bayesian Networks (BNs) and multivariate Gaussian and agent-based models [103].

In addition to these AI technologies, a cost-benefit evaluation addressing life cycle net present value (NPV) can be applied to support energy consumption of building intelligence systems [101]. Specifically, to develop nearly zero energy buildings (NZEBs), energy consumption standards can adopt energy-efficient measures based on efficient thermal insulation systems, high-performance window systems (heat transfer coefficient, solar heat gain coefficient and window-to-wall ratio), good airtightness and fresh air heat recovery systems. Furthermore, NZEBs use various renewable energy technologies such as solar thermal systems, solar PV systems, ground source heat pumps (GSHP), air source heat pumps (ASHP) and wind power systems [136].

Intelligent controls with smart sensing and self-learning behaviors have been used for both *energy* and *comfort*. However, there are some interesting characteristics of *thermal comfort-ensuring behaviors*. For example, Cheng et al. [107] address human thermal comfort measurement using a contactless measurement algorithm and Peng et al. [114] developed a learning-based (using ANN) temperature preference control (LTPC) as an occupant-centric climate control system. Yoganathan et al. [111] introduce an optimal sensor placement strategy using clustering algorithms that optimize the number and location of sensing points. A recommender system using distributed sensing, context-awareness and ML can also be applied for personalized visual comfort [106], while a decentralized stochastic control using a Markov decision process can be used for comfort-ensuring behaviors [123]. An indoor localization system based on ANN and particle filters is also proposed for customized comfort service [128]. In addition to thermal and visual comfort, acoustic comfort has also been considered in smart environments [117,120].

In addition, there are three comfort control strategies—conventional methods, intelligent control and multi-agent-based modelling (MABM) techniques—which enable an intelligent BECM system [99]. To develop *comfort-ensuring CBs* in smart environments, intelligent building control systems have adopted computational optimized operational methods: occupant behavior modelling, and data collection, analysis and feedback. Modelling occupant behavior involves deterministic, stochastic and agent-based behavioral modelling techniques, while computational optimization is achieved by single-objective optimization (SOOP), multi-objective optimization (MOOP) and classical methods such as the weighted sum method (WSM) and evolutionary algorithms [99]. These AI methods and techniques are used to simultaneously optimize *energy and comfort-related behaviors* in buildings, supported by the *self-learning behaviors* discussed in the previous sections. This intelligent aspect of smart environments also links to *adaptive comfort behaviors* including psychological and physiological adaptation as well as behavioral adjustment [99].

A smart HVAC system should be a long-term research topic for smart environments, but recent studies adopt intelligent HVAC controls using real-time occupancy recognition [103,105,116], an MPC [119,132], a MOOP method [117], a fuzzy supervised neural-control (FSNC) [126] and hybrid learning [124]. These *indoor comfort-ensuring CBs* also require sensing systems. For example, to determine an occupant’s thermal comfort preference, temperature and humidity, velocity and heart rate and skin temperature sensors can be used in the building system. In contrast, individual visual comfort can be determined using photometric and mobile pupilometer sensors [102].

The final observation of this last dataset involves safety, design and maintenance behaviors in smart environments [129]. *Safety* research is concerned with reducing the risk of harm for occupants, although it can consider crowd safety [131], privacy and security issues [102] and health and safety requirements [137]. *Design* (e.g., architectural, electrical, mechanical or layout design) can be improved by the integration of automation and control systems in a building [129]. Thus, from a design perspective, smart homes continue to be a research topic [113,138,139] along with façade design [137,140,141,142,143]. Automated adaptive façade functions [140] and occupant–facade interaction [141] not only present *energy and comfort* related behaviors, but also impact on building design. Furthermore, solar PV systems [136], smart materials [137] and phase change materials [143] can be investigated for intelligent building design. Recently, intelligent building design is linking to its life-cycle *maintenance*, significantly supported by BIM [100,127,144]. BIM also supports indoor navigation [127] and intelligent disaster prevention [144]. In addition, the management and maintenance of an intelligent building can adopt cognitive facility management [145], real-time digitalization [105] and even autonomous robots [146].

## 5. Discussion

### 5.1. Sensing Behaviors

**Figure 1:**
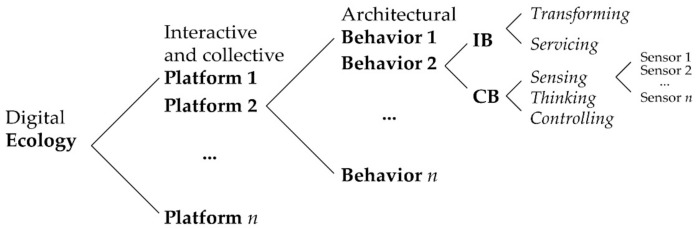
Spatial and informational hierarchy of smart components in the digital ecosystem.

While this paper has largely addressed architectural IBs and CBs in smart and responsive environments, the identification of specific sensing behaviors is also important for developing smart spaces, because it supports the transformation of architecture into a digital, dynamic platform. There is a clear spatial and informational hierarchy between sensing behaviors, architectural behaviors (IBs and CBs) and smart environments (interactive and collective platforms) in the digital ecosystem, as shown in Figure 1. Thus, an understanding of sensing behaviors provides an essential foundation for the construction of a smart environment. In a similar, albeit inverse way, this review on the IBs and CBs of smart environments contributes to a better understanding of sensing behaviors used in the built environment. For example, environmental sensing behaviors [59,63] involve not only temperature and humidity sensors, but also capture diverse sensory information such as noise and air flow, corresponding to the IBs that the environments exhibit. Smart sensing behaviors, furthermore, enable complex energy controls as well as optimized environmental services by way of sensor fusion. Smart sensing behaviors involving wearable sensors and identification technologies such as RFID can also be used for precise customized IBs and CBs. Following this logic, smart environments can be regarded as sensor-based platforms. Nonetheless, these sensing behaviors are not fully investigated in this review. Thus, a systematic review on sensing behaviors should be considered for future research.

Several articles in the last dataset, intelligent building, provide some important frameworks for a future study about sensors. For example, the data-driven control of an intelligent building relies on data collected from sensors [110]. Thus, intelligent buildings in the residential, office and retail sectors adopt various types of sensor systems for better environment control [103]. For example, real-time electricity data can be collected from smart meters [113] and an energy consumption model can be developed using occupancy monitoring solutions [147]. An intelligent EMS uses micro-climatization by smart sensor systems and real-time digitalization, learning user behaviors [105]. The *self-learning behavior* based on *smart sensing behavior* should be an essential component of a BECM system. Significantly, Dong et al. [102] highlight three categories of sensing systems for building operation: (i) occupancy sensing systems, (ii) environmental sensors and (iii) other sensors (wearable and IoT-based sensors). The first and third categories capture occupant behaviors and patterns, while the second category is used for understanding indoor environmental characteristics. The first includes image-based sensors, motion sensors, radio-based sensors and threshold and mechanical sensors, determining occupancy [102]. For example, passive infrared (PIR) sensors, ultrasonic and microwave Dopplers and ultrasonic ranging are motion sensors, while RFID, WiFi or Bluetooth, global positioning system (GPS) and ultra-wideband (UWB) are radio-based sensors [102]. In contrast, environmental sensors include sensors for temperature, humidity, air velocity, photometric, CO_2_, volatile organic compounds (VOC) and particulate matter (PM). Furthermore, Dong et al. [102] present some useful applications of sensors in the built environment. For example, for *energy saving behaviors*, a BECM system can use a CO_2_ sensor, chair sensor, PIR sensor, photometric sensor and smartphones or IoT applications. As discussed above, research of this type about sensing systems should also be useful for constructing different smart spaces because sensing is the most fundamental CB in smart environments.

### 5.2. Key Behaviors of Responsive Architecture

All four keyword combinations (leading to specific datasets) used for the systematic literature review in this paper have, to a certain extent, been used interchangeably in architectural research. This paper, however, reveals some differences between them, in part because their origins and concerns are historically different. For example, “kinetic architecture” highlights various aspects of *kinetic* hardware and its movement, while “adaptive architecture” is more focused on *structural* and *evolutionary* aspects of behaviors. In contrast, “intelligent building” research is strongly limited to energy and comfort related behaviors. Table 2 summarizes key behaviors presented across the four subjects and datasets. As this research has classified and developed a new understanding of kinetic, adaptive and intelligent architecture in terms of interactive and collective platforms, the table also identifies key IBs and CBs of each subject. *Climate-responsive behaviors* are widely exhibited by “responsive architecture”, “kinetic architecture” and “adaptive architecture” in terms of *mechatronic* and *origami-based IBs*. In contrast, *structural adaptive behaviors*, *energy-optimizing behaviors* and *comfort-ensuring behaviors* frequently happen in “adaptive architecture” and “intelligent building”. Collectively, “kinetic architecture” is closely linked to “responsive architecture”, while “adaptive architecture” connects to both “responsive architecture” and “intelligent building”. The last two key behaviors, *energy-optimizing behavior* and *comfort-ensuring behavior*, have adopted more advanced CBs such as intelligent control and smart sensing than the others. Thus, the application of advanced CBs to the other key behaviors can improve the kinetic performance of smart environments.

Interestingly, *biomimetic behaviors*, which highlight self-actuating IBs without any CB, have recently been implemented in smart environments, and especially those encapsulated under the titles “responsive architecture”, “kinetic architecture” and “adaptive architecture”. However, considering recent popular, practical interest in bio-inspired design and biomimicry, the evolutionary behaviors should be further explored for the future application of “living architecture”. Importantly, smart materials can express five types of IBs: immediacy, transiency, self-actuation, selectivity and directness. Immediacy behavior responds in real-time, and transiency responds to more than one environmental state [37]. Self-actuation refers to internal intelligence. The selectivity response is discrete and predictable and the directness response is local to the “activating” event [37]. In addition, smart material systems in architectural practice can exhibit three types of behaviors: (i) structural, (ii) climate and energy and (iii) architectural. Structural behaviors include safety monitoring and self-healing properties, while climate and energy behaviors use latent heat storage, adaptive daylight systems and energy harvesting. Lastly, architectural behaviors involve lighting and displaying technology, space division, aesthetic and entertainment adaptations and self-cleaning technology [86,148].

The discovery of emerging architectural behaviors beyond key behaviors in Table 2 can significantly impact on the development of creative smart environments. For example, the classification of kinetic systems presented in two review papers [44,74] can further support the characterization of smart environments in terms of kinetics in architecture. Fox and Yeh [44,149] also classify six *control behaviors* for kinetic systems: internal control, direct control, indirect control (computer control via sensor feedback), responsive indirect control, ubiquitous responsive indirect control and heuristic responsive indirect control. These additional classifications and smart material systems can contribute to exhibiting new IBs and CBs in architecture.

### 5.3. Interactive and Collective Platform

This paper has proposed a way of viewing smart environments as digital, dynamic platforms, characterized by various IBs and CBs. There are precedents for this, with interactive and collective platforms already being realized in multi-media artworks. Lee et al. [28], for example, suggest two types of platforms: *mobile platforms* that use a MAR platform as a collective interface and *situated platforms* that use ubiquitous sensor networks to collect data from users and respond to their presence (*self-organizing behavior*). Particularly, *situated platforms* are developed by *mechatronic behaviors* and intelligent controls with smart sensing and even *self-organizing* or *self-learning behaviors* discussed in the previous sections, but they commonly use large “situated displays” in outdoor or public spaces. Recent interactive media installations include *Discussions in Space* [150], *Sapporo World Window* [151], *SMSlingshot* [152], *Smart Citizen Sentiment Dashboard* [153], *City-Share* [154] and *iFloor* [155]. With new (e.g., foldable, bendable and rollable) display technologies, this *simulated* architecture can support the more interactive, collective and immersive behaviors blurring or merging both physical and digital spaces. Although Negroponte identified two types of responsive behaviors (reflexive and simulated), *simulated behaviors* are only addressed in a few articles. For example, Meyboom et al. [63] introduce an interactive landscape and a virtual bridge, while Park [60] deals with a virtual–physical prototyping environment. However, the *simulated behaviors* can use wearable devices and/or large displays that place the users into a virtual or augmented realm. Thus, they can be easily implemented in any smart space, considering “reality-virtuality continuum” ranging from AR to augmented virtuality [156]. Thus, this *simulated behavior* should be further investigated as a key behavior of creative smart platforms in the digital ecosystem.

In summary, as architecture becomes a form of service or behavior, the surroundings can be conceptualized as software rather than hardware. Thus, “process replaces product in importance, just as system supersedes structure” [157]. Inhabitants of the digital ecosystem actively or pervasively participate in the interactive process of smart environments, which conveys self-organizing architectural behaviors. This type of IB can impact on individuals, communities and cultures linked through myriad sensory devices in a dynamic environment, developing “architectural responsiveness” [158]. Such IB relies on ambient recognition and intelligence to collect human activities and environmental data [159], presenting an augmented space, or so-called “*sensponsive architecture*” [43]. In addition, smart environments do not just enable individuals’ interactions with their surroundings, but also support social CBs with the physical environment. Data recording CBs in the digital ecosystem has been linked to *reflexive* collective intelligence [160]. Thus, IBs and CBs captured in this paper are essential for future architecture because they are central to understanding smart environments as continuously evolving, digital platforms.

## 6. Conclusions

This research has investigated key behaviors in “responsive architecture” and three inter-related subjects, “kinetic architecture”, “adaptive architecture”, and “intelligent building”. All four subjects share some key behaviors, but each has different specific IBs and CBs (Table 2). In other words, some limitations of each subject can be complemented by the IBs and CBs of the other subjects. In addition, the combination of IBs and CBs identified in this paper can create different controlled or programmed effects, but its capacity remains an open question for a future interactive and collective platform to explore. Thus, this paper contributes to understanding and designing responsive artificiality that is relevant to design related transdisciplinary fields.

This paper has addressed four subjects and their key behaviors, which covers most of the characteristics related to “responsive architecture”. However, other subjects—“smart architecture”, “flexible architecture” and “performance-based architecture”—might be able to exhibit the alternative IBs and CBs that are not uncovered in this paper. In addition, the three academic databases used for the systematic literature review might not cover all relevant architectural research. Thus, a future study is to focus on these methodological limitations. Nonetheless, it is proposed that the five key behaviors identified in this paper—climate-responsive, biomimetic, structural adaptive, energy-optimizing and comfort-ensuring—would still be dominant in the related subjects.

Through the research conducted in this paper, it is apparent that the smart environment is no longer a “hard” architecture, but it is a platform where various IBs and CBs are exhibited. Furthermore, an interactive and collective platform will evolve through the actions of occupants as well as environments. As an informative reference in this field of research, this paper contributes to characterizing and creating the intelligent platform in the digital ecosystem.

## Figures and Tables

**Table 1:** General characteristics of the four literature datasets.

Dataset (Subject)	Number of Articles	Top Three Dominant Journals (Number of Articles)
Responsive Architecture	25	*Architectural Science Review* (4), *International Journal of Architectural Computing* (4), *Automation in Construction* (2), *Frontiers of Architectural Research* (2)
Kinetic Architecture	14	*International Journal of Architectural Computing* (2), *International Journal of Space Structure* (2), *Mechanics Based Design of Structures and Machines* (2)
Adaptive Architecture	14	No dominant journal
Intelligent Building	173	*Energy and Buildings* (30), *Intelligent Buildings International* (23), *Automation in Construction* (9), *Building and Environment* (9)

**Table 2:** Key behaviors of smart environments.

Key Behavior	Featured IBs	Featured CBs
Climate-responsive behavior	Mechatronic behaviors, Origami-based behavior	Automated control behavior, Environmental sensing behavior, Self-organizing behavior
Biomimetic behavior	Self-actuating behavior, Material-dependent behavior, Hygroscopic behavior, Evolutionary behavior	-
Structural adaptive behavior	Mechatronic behaviors, Origami-based behavior, Translational motion behavior	Self-organizing behavior, Self-learning behavior, Environmental sensing behavior
Energy-optimizing behavior	Energy efficiency or saving behavior, HVAC and lighting behavior	Intelligent control behavior, Smart sensing behavior, Self-learning behavior
Comfort-ensuring behavior	Thermal comfort behavior, Visual comfort behavior, Adaptive comfort behavior	Intelligent control behavior, Smart sensing behavior, Self-learning behavior

## Data Availability

The data presented in this study are available on request from the corresponding author. The data are not publicly available due to the policy of research projects.
